# Global research on sufentanil use in anesthesiology from 2003 to 2023: a bibliometric analysis

**DOI:** 10.3389/fphar.2024.1412726

**Published:** 2024-09-26

**Authors:** Duoqin Huang, Zixin Luo, Xinyue Song, Kang Zou

**Affiliations:** ^1^ The First Clinical Medical College, Gannan Medical University, Ganzhou, China; ^2^ Department of Critical Care Medicine, The First Affiliated Hospital of Gannan Medical University, Ganzhou, China

**Keywords:** sufentanil, Web of Science, anesthesiology, bibliometric, visualization analysis

## Abstract

**Objective:**

The application of sufentanil of anesthesiology has become a popular research area. However, literature-based bibliometric analyses on sufentanil are limited. Therefore, this study aimed to review the application of sufentanil in anesthesiology, and evaluate the research status and trends in this field.

**Methods:**

We searched the SCI-Expanded, SSCI, and CPCI-S databases from the Web of Science core collection as data sources for articles published from 1 January2003, to 31 December2023, and bibliometric and VOSviewer software were used to visualize and analyze the literature in terms of authors, journals, countries, institutions, and their collaborative networks, as well as keyword networks.

**Results:**

Our analysis included 1,473 relevant publications on the application of sufentanil in anesthesiology. The overall number of publications is on the rise; the top three countries of study were China, the US and France; the top three universities that published relevant articles were Anhui Medical University, Capital Medical University and Zhejiang University; the largest number of publications focused on *Anesthesia and analgesia*; At present, the studies in this field mainly focus on the application scope, mode, and advantages; adverse reactions; and combined effects of sufentanil in combination with other drugs. The adverse factors for the use of sufentanil in anesthesiology and ways to improve its safety and efficacy are hot topics of research. Future research should explore the applicability of population and dose utilization, novel drug combinations, non-opioid adjuncts, and technological innovations.

**Conclusion:**

An increasing number of publications indicates that researchers are showing interest in the field of sufentanil use in anesthesiology, and ongoing research is at a relatively mature level. While the international community has established a strong foundation for cooperation, the cooperation among researchers, institutions, and countries needs to be enhanced. Simultaneously, efforts must be made to explore and strengthen personnel cooperation, expand the coverage of funding support, and improve the quality of the literature.

## 1 Introduction

Sufentanil is a newly synthesized potent opioid analgesic and a specific μ-opioid receptor agonist (Celleno, 1998). It has the advantages of a fast onset of action, strong analgesic effect, long duration of action, quick recovery from anesthesia, and ventilatory depression, and it can effectively suppress the stress response of patients during tracheal intubation (Ferouz and Norris, 1998; Thomson et al., 2000). It is a commonly used analgesic in clinical practice, associated with good hemodynamic stability and, sufficient myocardial oxygen supply (Lundeberg and Roelofse, 2011; Bovill et al., 1984). Notably sufentanil has fewers adverse effects than those of traditional opioid analgesics such as morphine and fentanyl (Madej et al., 1987; Sinatra et al., 1991). Sufentanil is approximately twice more lipophilic, crosses the blood-brain barrier more easily, has a higher plasma protein-binding rate, and has a smaller distribution volume than fentanyl (Flacke et al., 1985). In addition, after intravenous administration of sufentanil, as an opioid receptor agonist, it binds to plasma proteins in the human body, and is rapidly transported to the brain within a short period of time, ensuring the smooth circulation of blood, prevention of subdural hematoma, and providing an anesthetic effect (AuthorAnonymous et al., 1993). A recent meta-analysis showed that the incidence of agitation after anesthesia with sufentanil was lower, and the pain during the recovery period was less intense (Fu et al., 2023). Recent studies have shown that sufentanil inhibits cancer cell proliferation in a concentration-dependent manner and can be used for tumor treatment (Guan et al., 2022). However, during the induction of general anesthesia, sufentanil can lead to coughing, which can cause severe hemodynamic fluctuations and even be life threatening (Chen et al., 2021). Other studies have shown that sufentanil use occasionally results in respiratory depression during the postoperative recovery period (Dyer et al., 1992). The chemical and pharmacological effects of sufentanil have received considerable attention in recent years. Owing to its stable pharmacokinetics and strong analgesic properties, it is widely used in clinical treatments, such as the induction of general anesthesia, tracheal intubation, maintenance of anesthesia during surgery, and postoperative analgesia. Therefore, the adverse reactions to sufentanil should also be considered.

Research on sufentanil covers a wide range of topics and dimensions. Anesthesiology, as an independent discipline in clinical medicine, mainly focuses on the study of anesthesia, analgesia, resuscitation, and critical care medicine, and belongs to an interdisciplinary field of medicine. Anesthesiologists need to analyze multiple factors when administering anesthesia to patients and choose appropriate anesthetic drugs and methods to minimize the impact of anesthesia on patients. Bibliometric analysis is a comprehensive system that uses statistical methods to quantitatively analyze all data, and systematically review specific topics and analyze research hotspots and trends (Sugimoto et al., 2019). By mining data from large databases using search tools, we can analyze the patterns and explanations of unstructured knowledge and understand the development of a specific field in the global research community. Owing to these advantages, this method has been applied in multiple disciplines, including basic medicine (Akmal et al., 2020), public health (Tang et al., 2022), biomaterials (Zhang et al., 2019; Yang et al., 2015), and clinical pharmacology (Kong et al., 2023). The VOS viewer is a free Java-based software that analyzes literature data and constructs bibliometric networks and visualizations (van Eck and Waltman, 2010). Currently, given the rapid development of anesthesiology and extensive research on sufentanil and since review articles are often subjective analyses, traditional methods of literature review can no longer meet the demand for data analyses. Therefore, this study aimed to objectively, quantitatively, and systematically present the relevant research on sufentanil in anesthesiology through a bibliometric analysis. Using the VOS viewer software, CiteSpace software, and bibliometric websites, etc., publication trends, literature keywords, authors, institutions and their collaborations, and co-citations of keywords, can be visualized and analyzed to identify research trends and hotspots in the field. To our knowledge, this is the first study to conduct a bibliometric analysis of classic articles of on sufentanil, aimed at, helping clinical practitioners and researchers understand the research hotspots and development directions in this field.

## 2 Methods

### 2.1 Search strategy and data source

**FIGURE 1:**
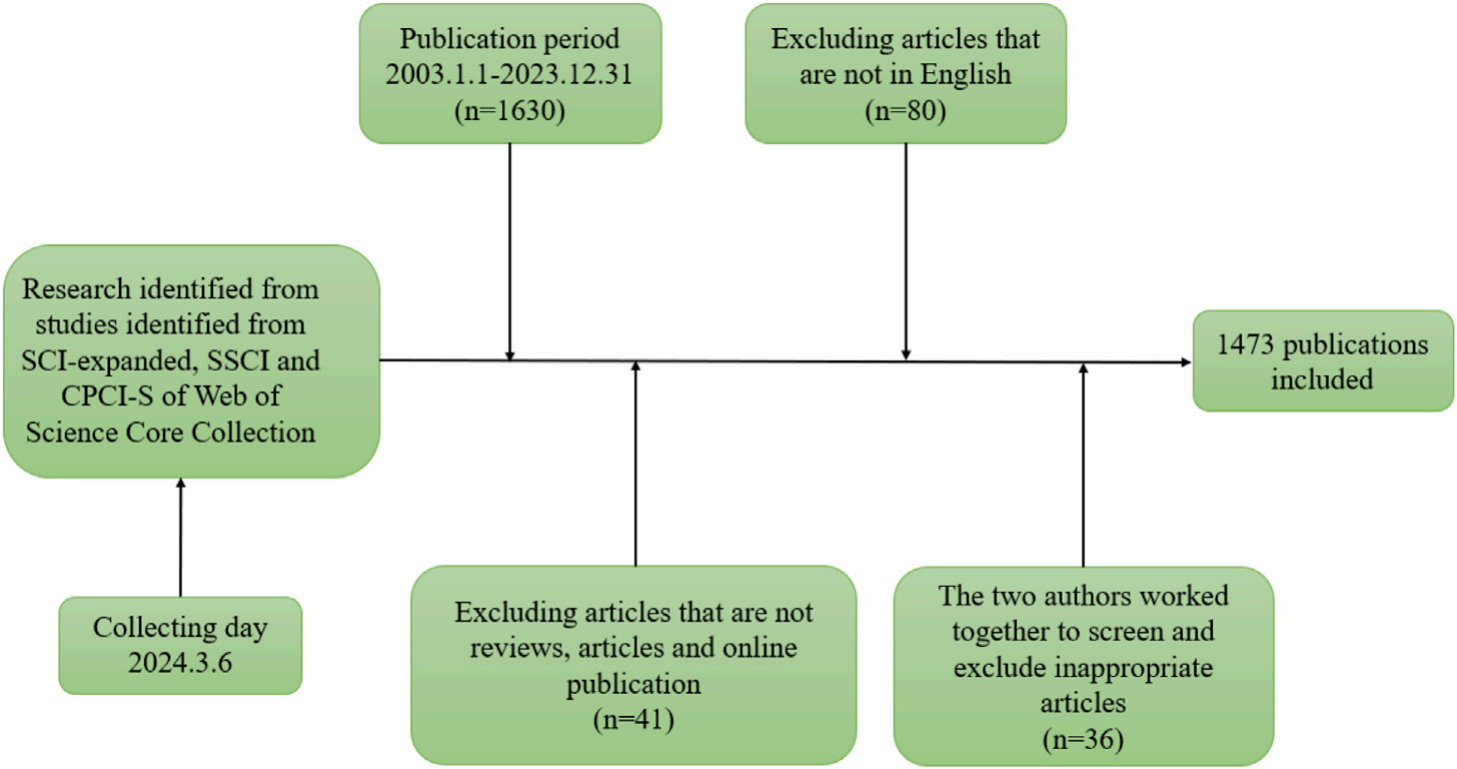
Flow chart of data source retrieval.

The Web of Science (WOS) is a multidisciplinary, international database renowned for its comprehensive collection of scholarly literature, recognized as an authoritative source in both the natural and social sciences. For this study, we utilized the WOS database, specifically targeting the SCI-Expanded, SSCI, and CPCI-S citation indexes within its core collection. Employing an advanced search strategy incorporating Boolean logic and relevant keywords, we crafted a search expression to pinpoint pertinent literature: (((((TS=(sufentanil)) OR TS=(sulfentanil)) OR TS=(sufenta)) OR TS=(sufentanilum)) OR TS=(Sufentanil Citrate Injection)) AND (((((((TS=(anesthesia)) OR TS=(anaesthesia)) OR TS=(anesthesiology)) OR TS=(anaesthesiology)) OR TS=(anesthetic)) OR TS=(anaesthetization)) OR TS=(autoanesthesia)). To further refine the search scope, we limited the publication date from 1 January 2003, to 31 December 2023, and the document types to “articles, reviews, and online publications (early access),” with the language set to “English.” To ensure the quality of the literature, two authors independently extracted basic information, reviewed, and screened the selected articles. Non-academic journal articles were excluded, as well as duplicates and irrelevant literature. The two authors had a 98% agreement in the screening process, and any remaining uncertain articles were discussed with a third author. The final analysis encompassed 1,329 articles. A flowchart illustrating the literature selection and data collection process is presented in Figure 1, with the search executed on 6 March 2024.

### 2.2 Data transformation and study methods

The selected literature will be exported in two formats: “plain text file” and “tab-separated file.” Bibliometrix, VOSviewer, and Citespace software will be used to perform statistical analysis and overview of the literature in terms of publication count, authors, countries, affiliations, citation status, and keywords. Descriptive statistical analysis of publication countries, affiliations, and journals will be conducted using Excel spreadsheets. Citespace 6.2.4 and VOSviewer 1.6.19 will be used to visualize and analyze the collaboration network, keywords, burst terms, and other aspects of the literature. Bibliometrix will be utilized for its aesthetic and detailed plotting features to analyze the author network, country network, affiliation network, and journal network. When conducting core analysis on authors, countries, and affiliations, we will employ Price’s theorem for estimation. Price’s theorem states that in a research field, the number of core authors with high productivity is approximately equal to the square root of the total number of authors. Through further derivation, the formula for the minimum publication count of core authors in a field is: M ≈ 0.749×(N_max_^1/2^), where Nmax represents the publication count of the most prolific scholar and M denotes the minimum publication count for core authors. This formula can be used to determine the core author group in the field, i.e., authors with publication counts greater than M are considered core authors in the field.

## 3 Result

A total of 1,473 articles published by 6,238 authors from 1,533 institutions in 63 countries were analyzed in this study. These articles were published in 355 journals, with a total of 29,163 citations from 4,505 journals. Among them, there were 1,372 research articles (93.14%), 101 review articles (6.86%), and included online published articles. As shown in Figure 1.

### 3.1 Analysis of the publications

**FIGURE 2:**
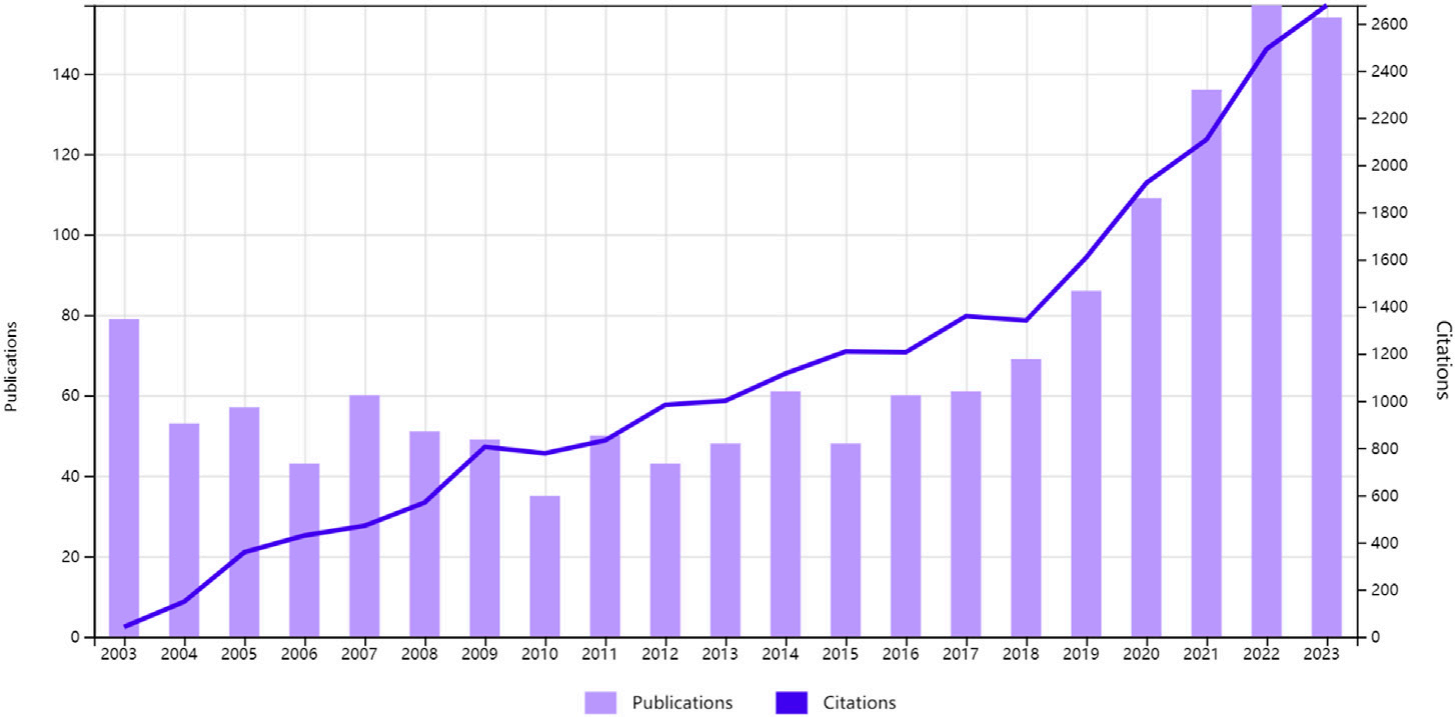
Annual publication volume from 2003 to 2023.

Figure 2 illustrates the retrieval of 1,630 articles from the WOS database, with 1,473 English-language articles spanning 2003 to 2023 selected for our analysis. The data reveal an upward trajectory in publication volume over the two-decade period. In 2003, 79 articles were published, accounting for 5.36% of the total, whereas the peak was reached in 2022 with 155 articles, representing 10.52% of the total—a 96.20% increase. The publication trend from 2003 to 2022 can be delineated into two phases: the first, from 2003 to 2014, showed a stable annual output without significant fluctuations; the second, from 2015 to 2023, witnessed a marked acceleration in publication rates. In 2015, there were 48 publications, which increased to 155 publications by 2022, more than tripled the initial count. The average annual growth rate during this period was 13.375, with an average annual growth rate of 27.86%. This trend underscores the ongoing potential for innovation within the field, warranting further scholarly exploration. Additionally, the escalating citation metrics suggest growing academic and professional recognition of the research domain.

### 3.2 Analysis of the countries

**FIGURE 3:**
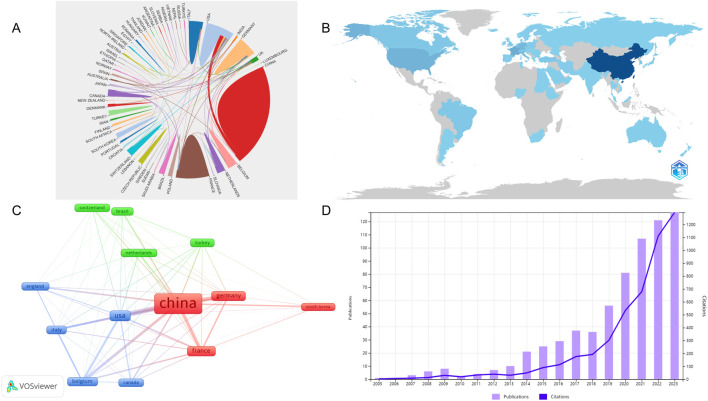
Analysis of the countries in global research on sufentanil used in anesthesiology. **(A)** Map of collaboration networks across all countries; **(B)** The visualization map of the published national cooperative geographic network; **(C)** Map of collaboration networks across core countries; **(D)** Annual Chinese publication volume from 2003 to 2022.

A total of 1,473 publications on sufentanil in anesthesiology were identified from 63 countries in the WOS database. China led with the highest publication count, totaling 674 articles (45.76%), followed by the United States with 162 (11.00%) and France with 130 (8.83%). In citation metrics, China also topped the list with 4,906 citations, succeeded by the United States and Germany. Among the top 10 publishing nations, the United Kingdom, Canada, and Germany exhibited the highest average citations per article. Detailed statistics are presented in Table 1. We believe that the number of published studies on sufentanil in anesthesiology in China partly maintains the number of published articles worldwide, so we separately draw the number of published articles in China from 2003 to 2022, as shown in Figure 3D. Similarly, we found that although China had the highest publication count, its academic influence in the field of sufentanil research in anesthesia was lower than that of other Western countries. This was mainly reflected in a lower average number of citations per article (=7.28). Figure 3A illustrates the international collaboration network, where color differentiation denotes distinct countries, and the thickness of the lines between nodes correlates with the volume of collaborative publications. China has the most robust and extensive network, closely followed by the United States. For a geographical perspective on collaboration, Figure 3B presents a graph with darker shades indicating higher publication counts and deeper lines signifying stronger collaborative ties. Figure 3C delineates the network of core publishing countries, identifying 13 key nations based on Price’s theorem, predominantly forming stable collaboration clusters centered around China. Notably, there are pronounced collaborative links between China and both the United States and Germany.

**TABLE 1:** Publications in the 10 most productive countries.

Ranked by TP	Country	TP	TC	TC/TP	Proportion(%)
1	China	674	4,906	7.28	45.76
2	United States	162	3,819	23.57	11.00
3	France	130	3,193	24.56	8.83
4	Germany	124	3,322	26.79	8.42
5	Belgium	74	1971	26.64	5.02
6	Italy	64	1,392	21.75	4.34
7	Turkey	43	749	17.42	2.92
8	Netherlands	41	1,024	24.98	2.78
9	Canada	40	1,237	30.93	2.72
10	England	39	1,167	29.92	2.65

TP, total publications; TC, total citations.

### 3.3 Analysis of the organizations

**FIGURE 4:**
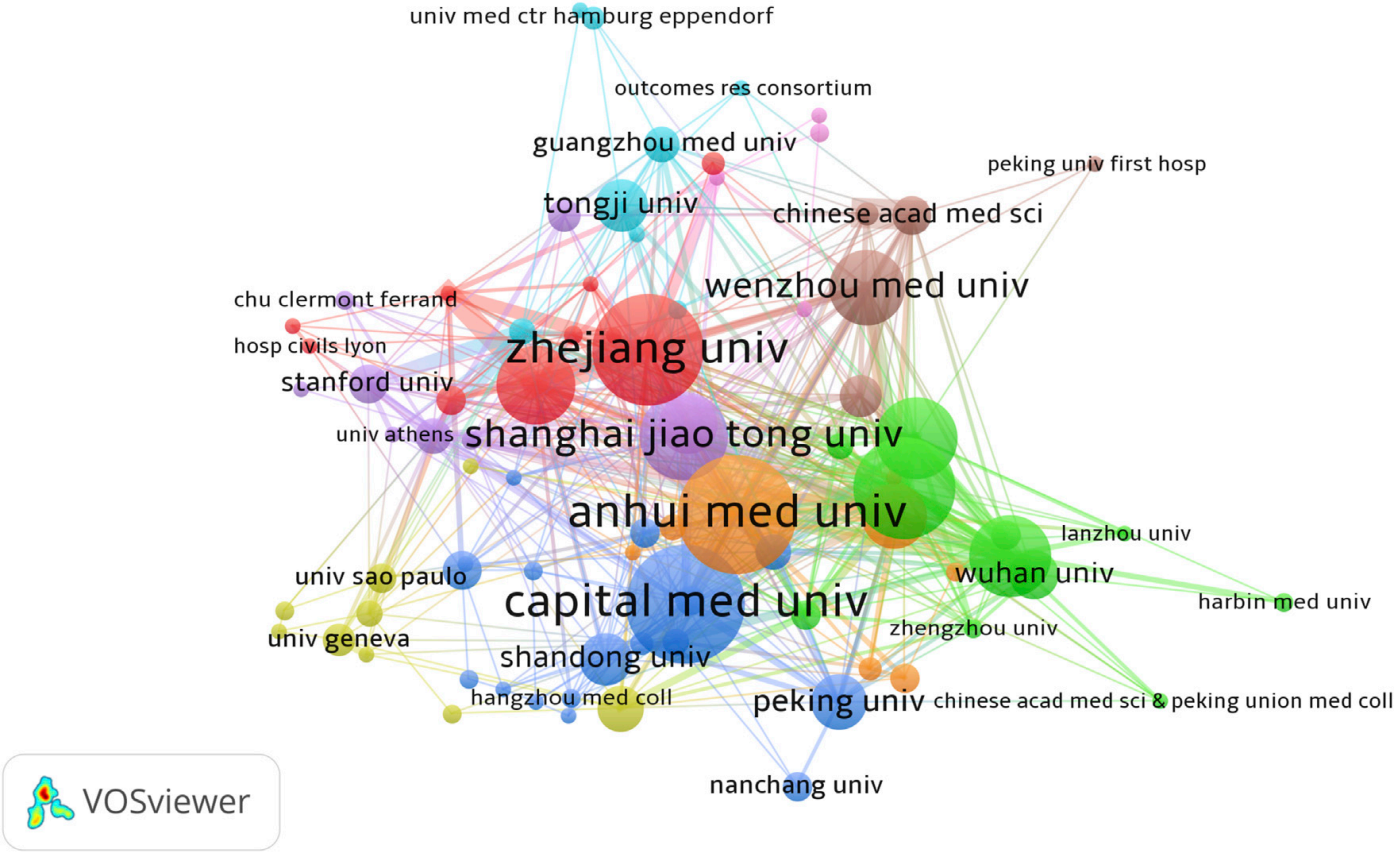
Map of cooperation network of core issuing organizations.

Statistics showed that a total of 1,533 research institutions participated in the study of sufentanil in anesthesiology. Notably, all of the top ten most prolific institutions are Chinese, underscoring the significant engagement and commitment of Chinese academia to sufentanil research. Anhui University, Capital Medical University, and Zhejiang University emerged as the leading institutions, with 36, 36, and 34 publications respectively (refer to Table 2 for details). Among these, Zhejiang University garnered the highest citation count, with 374 citations, and also led in terms of average citations per publication, at 11.00 times, closely followed by Fudan University with an average of 10.35 citations. Despite these achievements, the global average citation impact remains modest, indicating a need for more influential publications. Out of the 1,533 institutions, 83 with more than five publications were identified as core contributors. The collaboration among these institutions is characterized by a multi-core dynamic, with strong and intimate cooperative ties, as visualized in Figure 4. This network of core institutions suggests a robust and interconnected research community that is pivotal to the advancement of the field.

**TABLE 2:** Publications in the 10 most productive organizations.

Ranked by TP	Organizations	TP	TC	TC/TP	Proportion(%)	Country
1	Anhui Medical University	36	318	8.83	2.44	Anhui Province, China
2	Capital Medical University	36	265	7.36	2.44	Beijing, China
3	Zhejiang University	34	374	11.00	2.31	Zhejiang Province, China
4	Fudan University	31	321	10.35	2.10	Shanghai, China
5	Shanghai Jiao Tong University	27	172	6.37	1.83	Shanghai, China
6	Sichuan University	25	221	8.84	1.70	Sichuan Province, China
7	Huazhong University of Science and Technology	25	174	6.96	1.70	Hubei Province, China
8	Jiaxing University	24	211	8.79	1.63	Zhejiang Province, China
9	Wenzhou Medical University	23	153	6.65	1.56	Zhejiang Province, China
10	Nanjing Medical University	20	155	7.75	1.36	Jiangsu Province, China

There is an overlap in the output of papers across research organizations due to co-publications.

TP, total publications; TC, total citations.

### 3.4 Analysis of the journals

**FIGURE 5:**
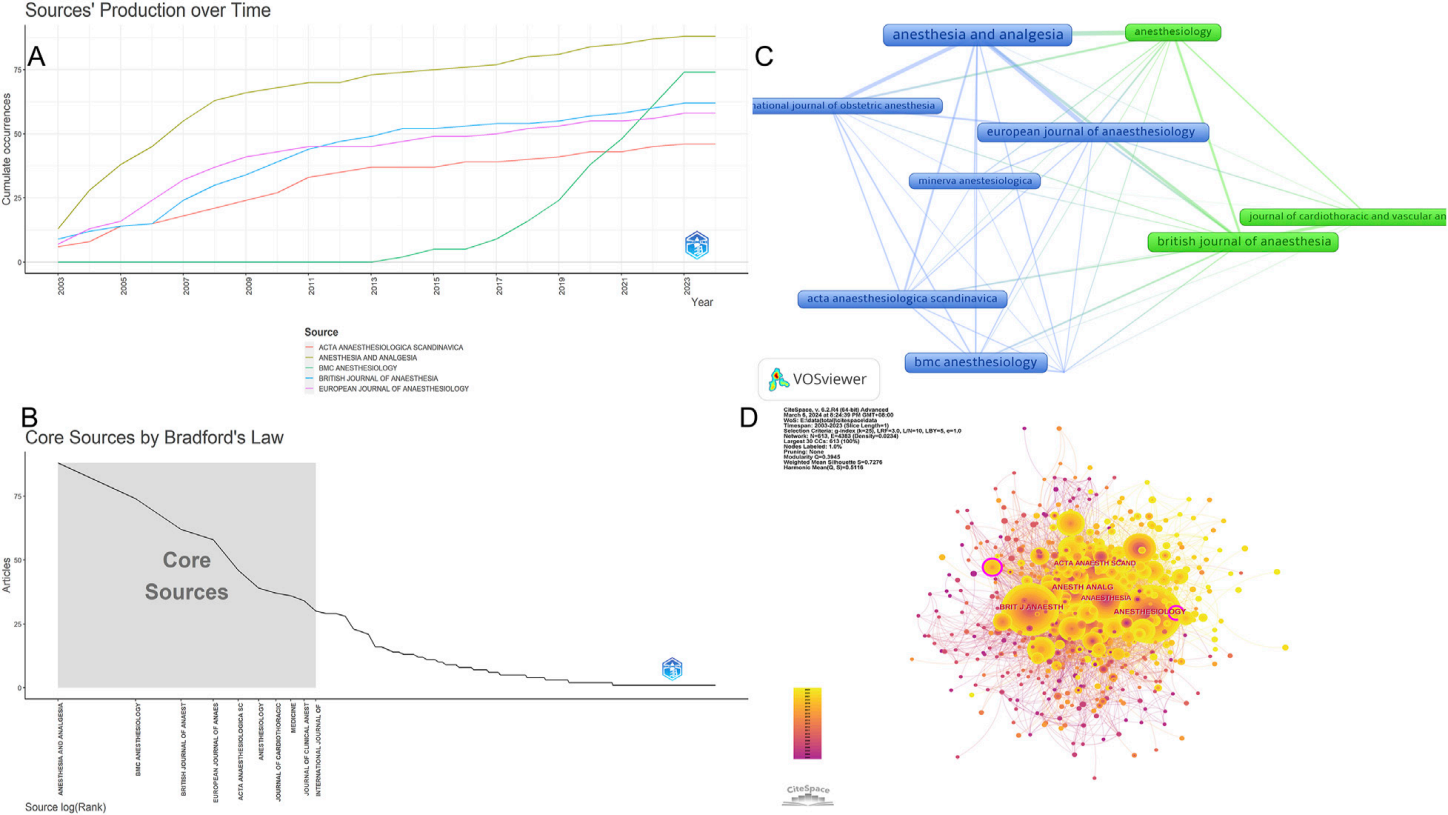
Analysis of the countries in global research on sufentanil used in anesthesiology. **(A)** Annual publication volume curve of the top 10 journals; **(B)** Map of collaboration networks across core journal; **(C)** Map of core journal; **(D)** Map of collaboration networks across all journal.

From 2003 to 2023, a total of 1,473 records were retrieved and published in 355 journals. Table 3 presents information on the top ten journals in terms of publication count, with a total of 504 articles accounting for 34.21% of the total publications. *Anesthesia and analgesia* had the highest publication count with 88 relevant publications, representing 5.97% of the total records. The second most significant journal was *BMC anesthesiology* with 74 publications. More than half of the top ten prolific journals analyzed were in the field of anesthesia. Based on total citations (TC), *Anesthesia and analgesia* had the highest TC (=3,289), followed by *Anesthesiology* (=2,376) and *British journal of anaesthesia* (=2,270). Additionally, among the top ten journals in terms of publication count, four were in Q1, with *B. journal of anaesthesia* having the highest impact factor (9.8). To gain insights into the current research trends in the relevant field, we plotted line graphs, as shown in Figure 5A, to analyze the annual publication growth trends of the top ten journals. Furthermore, using Bradford theorem, we identified core journals, as shown in Figure 5B and visualized their distribution in a network diagram, as shown in Figure 5C, for a more intuitive presentation. Meanwhile, in order to more comprehensively demonstrate the relationship between all journals, we drew the full-journals atlas using Citespace as shown in Figure 5D.

**TABLE 3:** Publications in the 10 most productive distribution.

Ranked by TP	Journal	TP	TC	TC/TP	IF/Q	Country
1	*Anesthesia and analgesia*	88	3,289	37.38	5.7/Q1	United States
2	*BMC anesthesiology*	74	586	7.92	2.2/Q3	England
3	*British journal of anaesthesia*	62	2,270	36.61	9.8/Q1	England
4	*European journal of anaesthesiology*	58	979	16.88	3.6/Q2	England
5	*Acta anaesthesiologica scandinavica*	46	1,225	26.63	2.1/Q4	Denmark
6	*Anesthesiology*	39	2,376	60.92	8.8/Q1	United States
7	*Journal of cardiothoracic and vascular anesthesia*	37	537	14.51	2.8/Q3	United States
9	*Medicine*	36	296	8.22	1.6/Q3	United States
8	*Journal of clinical anesthesia*	34	663	19.50	6.7/Q1	United States
10	*International journal of clinical and experimental medicine*	30	81	2.70	—	United States

TP, total publications; TC, total citations; IF, impact factor.

Since *International journal of clinical and experimental medicine* was not included in SCI, in 2023, there is no impact factor or zoning.

**FIGURE 6:**
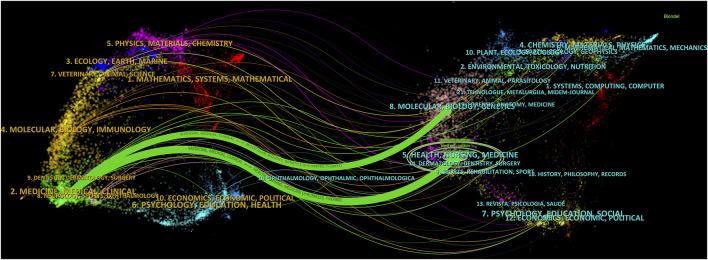
The dual-map overlay of journals on research of sufentanil used in anesthesiology.

Dual-map overlay is an effective tool in CiteSpace for revealing the disciplinary relationships of the records. In this map, the left side represents citing journals, while the right side represents cited journals. Citation waves are described on the base map, starting from the left side and pointing towards the cited side. It can be inferred that the cited side represents the forefront of research over the years, while the citing side represents their knowledge base. Therefore, we constructed a dual-map overlay, as shown in Figure 6, and used the z-score function to highlight strong connections, enhancing clarity and recognizability. Based on the overlay of dual-map journals, there are two main citation paths. Both paths originate from medicine and clinical research, one leading to public health, nursing, and pharmacy (z-score = 8.10), and the other extending to cell biology and genetics (z-score = 2.51). The cited articles are mainly related to anesthesia in the field of clinical medicine, clinical pharmacology, critical care medicine, cell biology in the field of basic medicine, and analytical biology. The published articles in journals cover clinical medicine, basic medicine, and pharmacy.

### 3.5 Analysis of the authors

**FIGURE 7:**
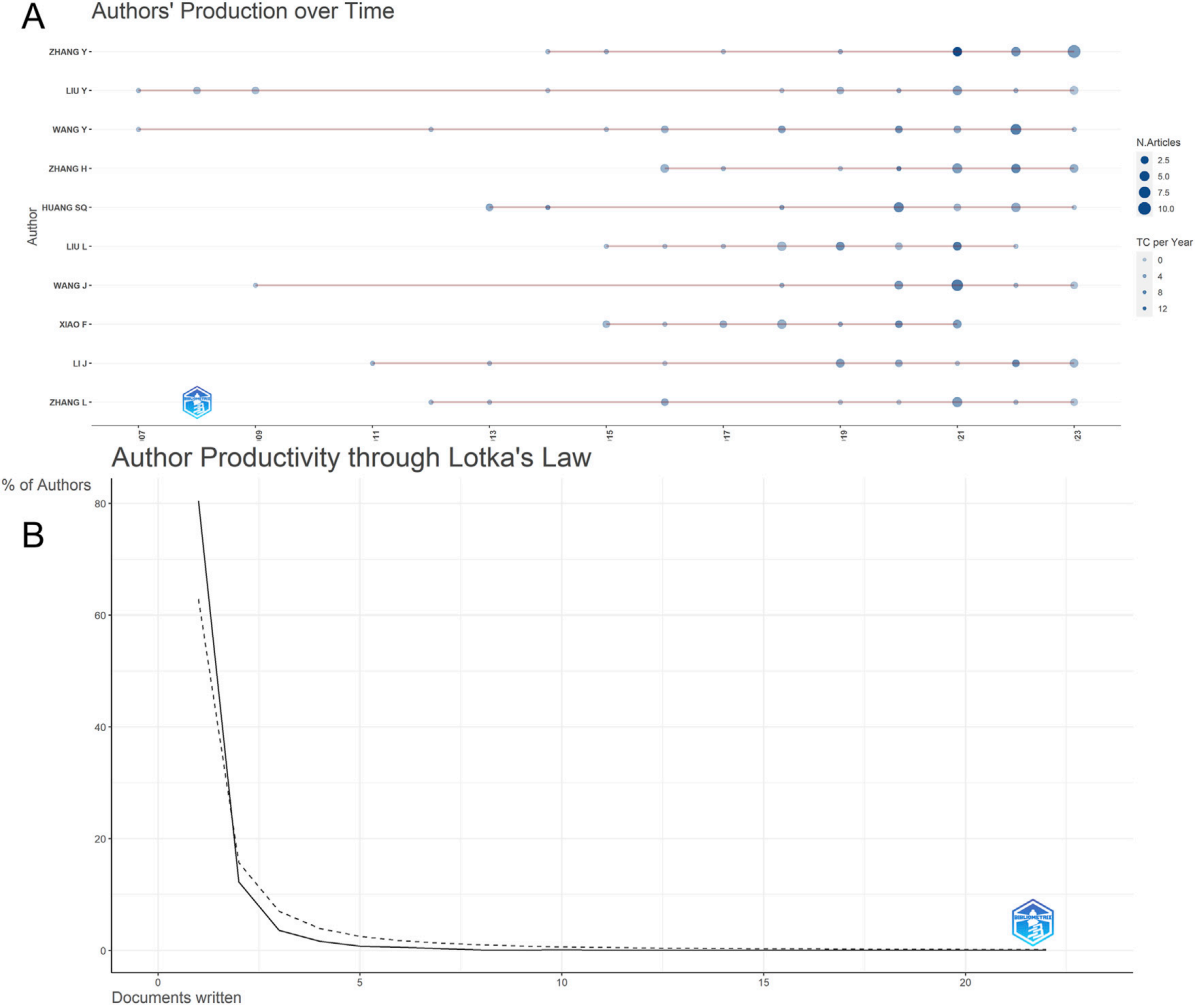
Analysis of the authors in global research on sufentanil used in anesthesiology; **(A)** Production over time of the top 10 authors; **(B)** Author productivity through Lotka’s law.

**FIGURE 8:**
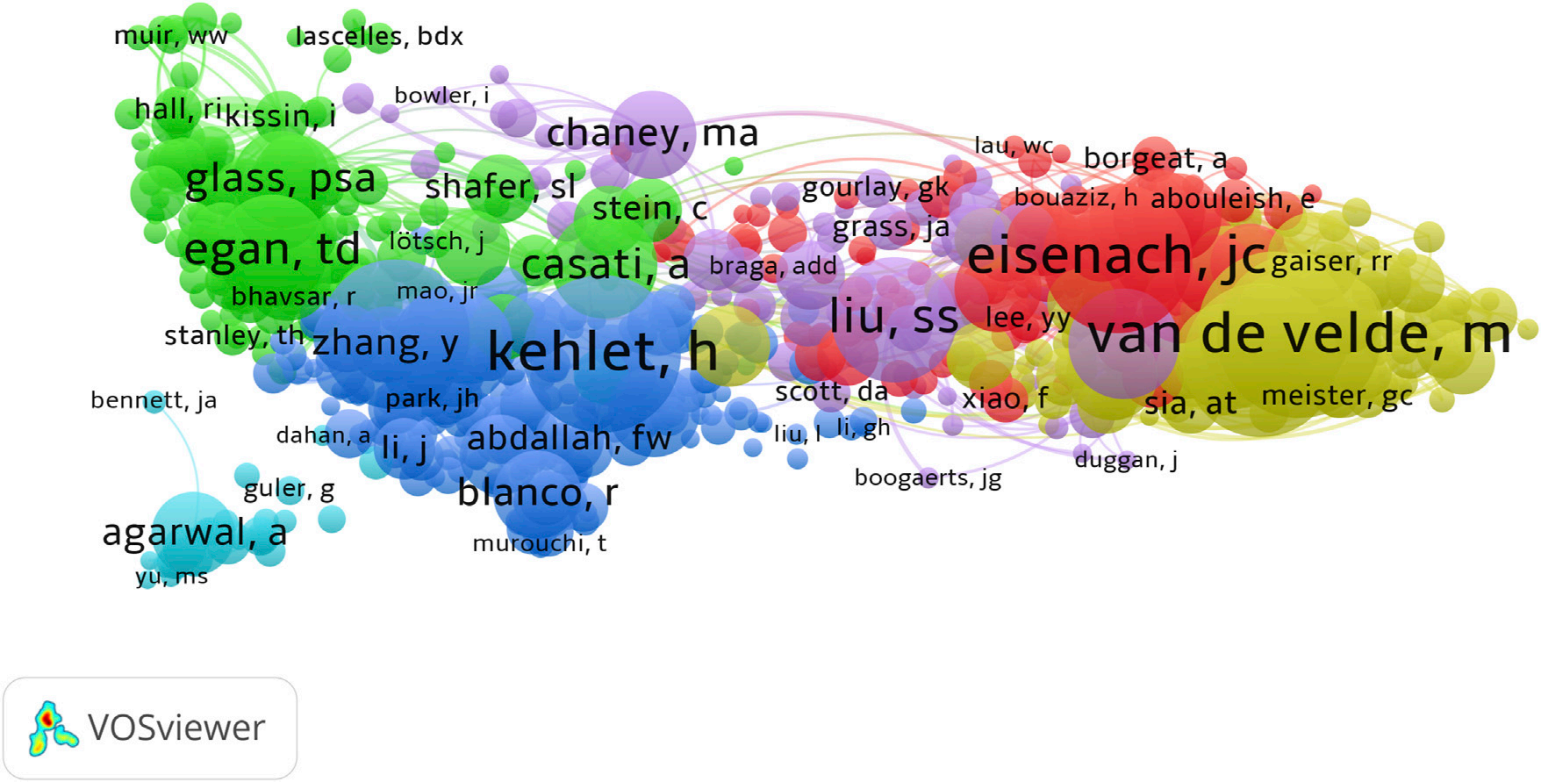
The visualization map of core co-cited authors.

The retrieved articles involved a total of 6,238 authors, with an average of 4.23 authors per article. ZHANG Y from China ranked first with 22 published articles, closely followed by WANG Y, LIU Y, and ZHANG H, each contributing 18 articles (Table 4). To explore the annual publication productivity of different authors, we depicted the average annual publication volume per author, as illustrated in Figure 7A. Simultaneously, employing Lotka’s Law graph (Figure 7B), results indicated that over 80% of authors contributed only one publication, while less than 1% of authors published five or more articles. Co-cited authors refer to a group of authors who are concurrently cited in two or more papers, forming a collaborative citation relationship indicative of contributions to the subsequent development of the field. In the context of research on the anesthesia application of sufentanil, a total of 20,832 authors were co-cited. By restricting authors with citations exceeding 8, the co-citation collaboration network among different authors in this field is depicted in Figure 8.

**TABLE 4:** Publications in the 10 most productive authors.

Ranked by TP	Authors	TP	TC	TC/TP	Proportion(%)	H_index	PY_start
1	Zhang Y	22	148	6.73	1.49	7	2014
2	Wang Y	18	169	9.39	1.22	8	2007
3	Liu Y	18	128	7.11	1.22	7	2007
4	Zhang H	18	130	7.22	1.22	6	2016
5	Liu L	16	117	7.31	1.09	8	2015
6	Huang SQ	16	200	12.50	1.09	7	2013
7	Xiao F	15	140	9.33	1.02	8	2015
8	Wang J	15	88	5.87	1.02	5	2009
9	Zhang YF	14	110	7.86	0.95	8	2007
10	Li J	14	115	8.21	0.95	6	2011

There is an overlap in the output of papers across research authors due to co-publications.

TP, total publications; TC, total citations.

### 3.6 Analysis of subject fields

According to the statistics of WOS, 1,473 articles on sufentanil cover 67 subject topics, and the number of topics continues to rise with time. The top five are anesthesiology, internal medicine, pharmacology, medical research experiments and clinical neurology (Table 5).

**TABLE 5:** Top 10 themes of psychological violence-related studies included in the WOS.

Rank	Subject areas	TP	Percentage(%)	Rank	Subject areas	TP	Percentage(%)
1	Anesthesiology	656	44.53	6	Surgery	73	4.96
2	Medicine General Internal	151	10.25	7	Cardiac Cardiovascular Systems	57	3.87
3	Pharmacology Pharmacy	136	9.23	8	Critical Care Medicine	54	3.67
4	Medicine Research Experimental	94	6.38	9	Obstetrics Gynecology	52	3.53
5	Clinical Neurology	85	5.77	10	Respiratory System	42	2.85

Due to cross-disciplinary phenomena, the same literature can be attributed to more than one discipline at the same time.

TP, total publications.

### 3.7 Analysis of the keywords

**FIGURE 9:**
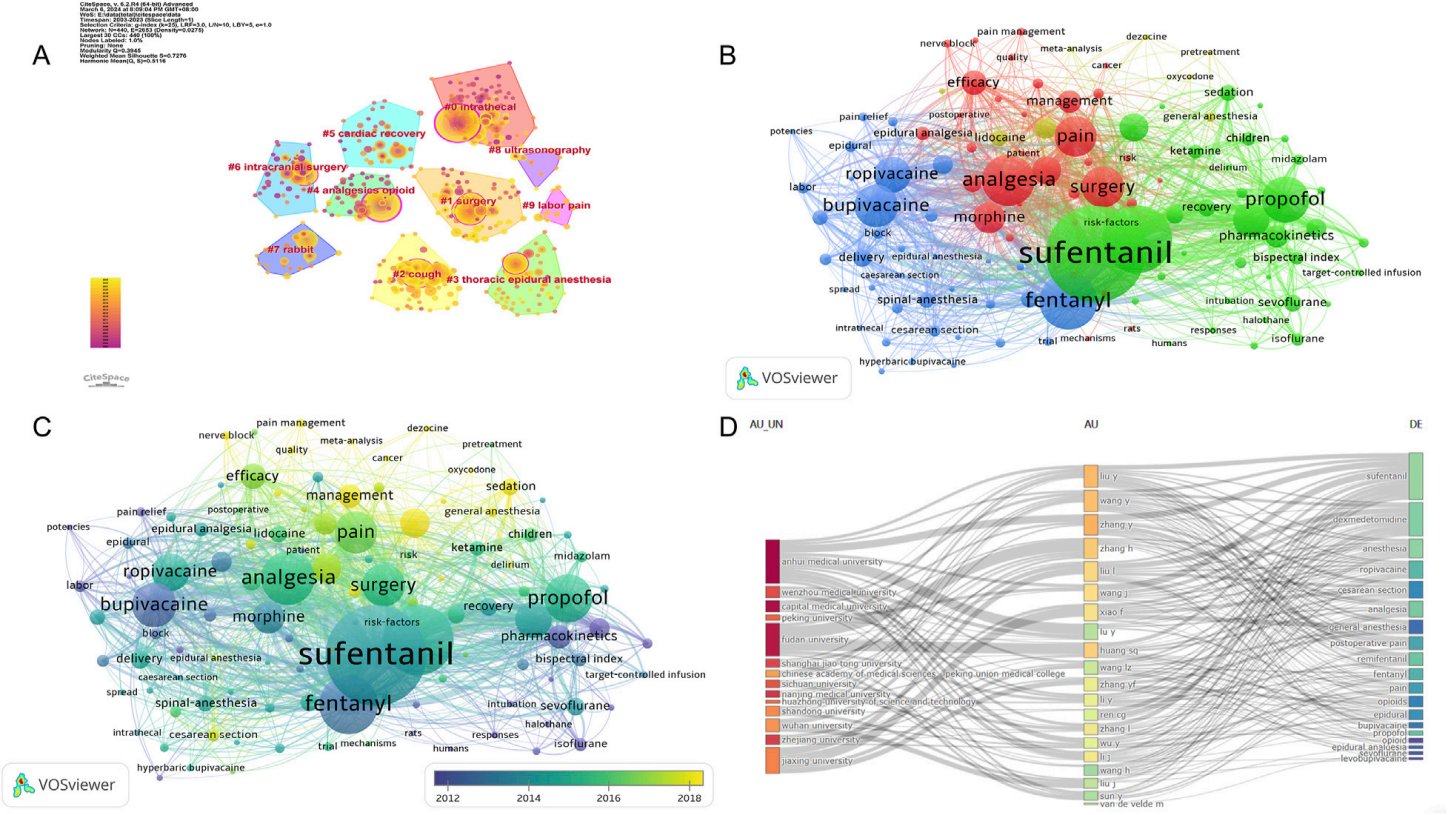
Analysis of the keywords in global research on sufentanil used in anesthesiology. **(A)** Research on the visualization map of keywords clustering; **(B)** The visual map of the core keywords; **(C)** The visualization of the core keyword hotpots and trends; **(D)** Three-fields plot of authors, affiliations, and keywords in major papers (Legend: Left-field: Affiliations; Middle field: Authors; Right-field: Keywords).

**FIGURE 10:**
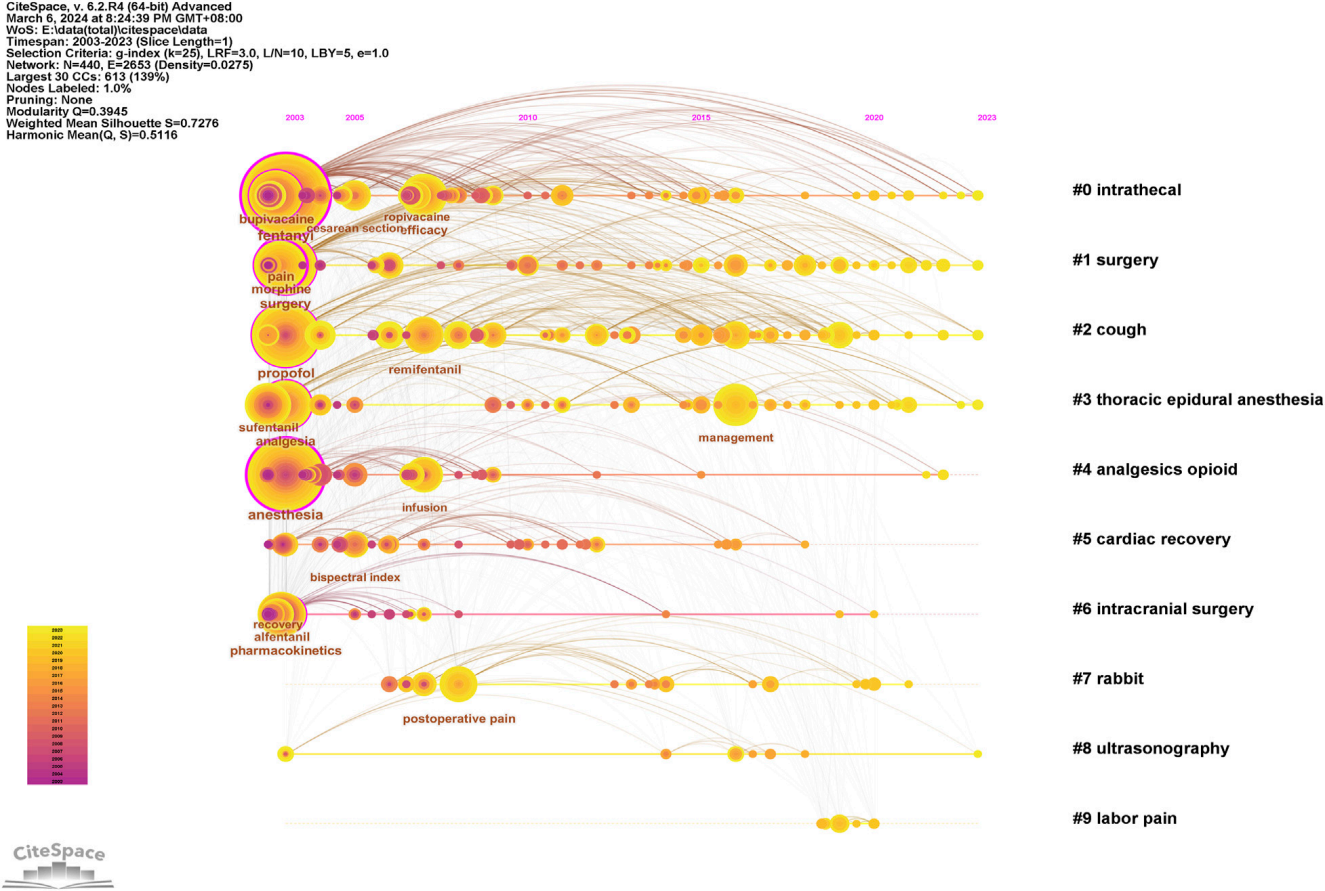
Timeline atlas of network clustering knowledge graph.

Keywords can intuitively reflect the themes and hotspots of a research field, with each selected keyword representing the essence and refinement of the subject matter. A total of 4,453 keywords were identified in the retrieved literature. To gain a clearer understanding of the specific circumstances of these keywords, the top 20 most frequent keywords are listed in Table 6, which represent the main hotspots of sufentanil research. To better summarize and categorize these numerous and complex keywords, we utilized CiteSpace software to create a keyword clustering map, as shown in Figure 9A. The results indicate that these clusters can be divided into 10 categories, each discussing various aspects of sufentanil’s pharmacokinetics, surgical applications, postoperative adverse reactions, and relationships with other anesthetics. More importantly, based on Price’s law, we constructed a network map of the main keywords with a frequency greater than 19 in the retrieved literature using VOSviewer software, as depicted in Figure 9B. This network contains 114 nodes, representing 114 core keywords. The larger the node, the higher the frequency of the keyword’s appearance, signifying the research focal points and hotspots in anesthesia that warrant our attention regarding sufentanil. Among these 114 keywords, they can be divided into four major clusters. Cluster 1 (Green) includes terms such as “pain management,” “analgesia,” “pain relief,” “nerve block,” and “ropivacaine.” This cluster focuses on the use of sufentanil in pain control strategies and its relationship with various other analgesics. Cluster 2 (Blue) is composed of keywords such as “spinal anesthesia,” “epidural anesthesia,” “general anesthesia,” “intubation,” and “bispectral index,” highlighting different anesthetic techniques and the monitoring of anesthetized patients, in which sufentanil may play a significant role. Cluster 3 (Red) is characterized by terms like “cesarean section,” “delivery,” “surgery,” and “postoperative,” emphasizing the application of sufentanil in surgical procedures and obstetrics, underscoring its importance in pain management during and after surgery. Cluster 4 (Yellow) includes keywords such as “pharmacokinetics,” “risk,” “patient,” and “recovery,” indicating that this cluster revolves around the pharmacological properties of sufentanil, considerations for patient safety, and the post-anesthetic recovery process, with a primary focus on side effects and adverse reactions induced by sufentanil, the most typical of which is coughing. Notably, within this research domain, numerous articles employ network meta-analysis to compare sufentanil with other commonly used anesthetics in clinical medicine, analyzing its advantages and identifying the most appropriate populations and diseases for its application. Furthermore, to clarify the primary research interests of key authors, we selected several individuals and constructed an institution-author-keyword tri-axial map, as depicted in Figure 9C. Concurrently, to better illustrate the evolution of research, we superimposed the keyword clustering map onto a timeline, generating a keyword timeline map, as shown in Figure 9D. The figure indicates that in recent years, Clusters 3 and 4 have received more research attention among the 114 core keywords. Additionally, to comprehensively examine the temporal dynamics of research hotspots, we created a timeline that includes all keywords, not just the core ones, as shown in Figure 10. The results reveal that from 2003 to 2023, different clusters exhibit distinct trends of change. It is evident that in recent years, research has primarily concentrated on adverse reactions and side effects following the use of sufentanil, such as cough analysis. Concurrently, sufentanil is recognized as a local anesthetic used in surgical and obstetric procedures.

**TABLE 6:** The top 20 high-frequency keywords.

Rank	Keywords	Occurrence	Rank	Keywords	Occurrence
1	Sufentanil	637	11	Morphine	163
2	Anesthesia	412	12	Dexmedetomidine	146
3	Fentanyl	334	13	Postoperative pain	145
4	Analgesia	298	14	Efficacy	123
5	Propofol	264	15	Pharmacokinetics	113
6	Bupivacaine	239	16	Management	111
7	Surgery	227	17	Double-blind	103
8	Pain	225	18	Infusion	92
9	Ropivacaine	195	19	Alfentanil	92
10	Remifentanil	190	20	Postoperative analgesia	86

### 3.8 The keyword burst analysis

**FIGURE 11:**
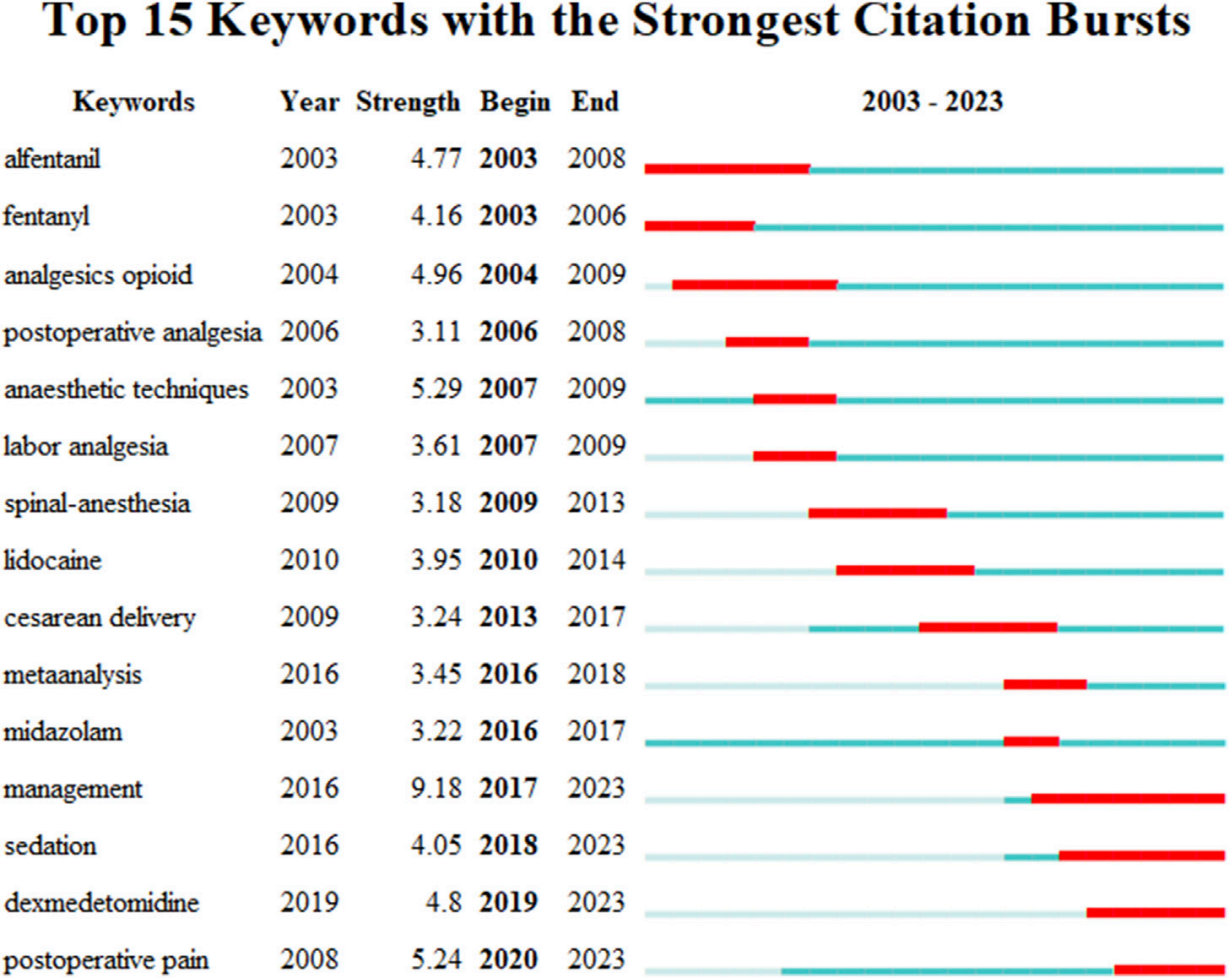
Research on the burst period and intensity of burst words.

Keyword burst analysis refers to the process of identifying keywords within a research field that have a rapidly increasing frequency over a certain period by analyzing the frequency-time distribution of keywords. This method can be utilized to recognize research hotspots, trends, and dynamic developments within the field. To gain a clearer understanding of the research hotspots regarding the application of sufentanil in anesthesia, we further employed CiteSpace’s burst function to analyze influential nodes within the research field across various time slices. We selected the top 15 keywords with the highest burst intensity, as depicted in Figure 11. The results indicate that the most intense burst keyword is “management” (with a burst intensity of 9.18), and related topics such as dexmedetomidine and opioid analgesics also exhibit high representation.

## 4 Discussion

### 4.1 Current research status

Sufentanil stands out as an efficacious and comparatively safe intravenous opioid analgesic, eliciting a quintessential opioid effect spectrum (Rosow, 1984). It demonstrates a significantly higher affinity for μ-opioid receptors, exceeding that of κ and δ receptors by more than an order of magnitude (Emmerson et al., 1994). The legacy of sufentanil originates in 1960 with Dr. Paul Janssen, the progenitor of Janssen Pharmaceutica—a subsidiary under the umbrella of Johnson and Johnson—who refined pethidine (meperidine), culminating in the synthesis of fentanyl. This novel analgesic offered superior potency and a more favorable side effect profile compared to morphine. The subsequent milestone was achieved in 1974 with the synthesis of sufentanil by Janssen. A decade later, in 1981, sufentanil made its debut in human anesthesia (Stanley, 1992). Its clinical utility is underscored by a suite of pharmacological attributes: a swift induction of anesthesia (Scholz et al., 1996), enduring analgesic efficacy (Gepts et al., 1995), the capacity to traverse the blood-brain barrier (van de Donk et al., 2018), minimal respiratory depressive effects (Bailey et al., 1990), stable hemodynamic profiles (Sebel and Bovil, 1982), expedited postoperative recovery (Monk et al., 1988), and a subdued impact on the stress response (Scott et al., 1991). These characteristics have propelled the adoption of sufentanil in various clinical settings, including anesthesia, postoperative analgesia, and ICU sedation.

Therefore, analyzing classic articles in this field is of great significance for understanding the clinical use of sufentanil. However, currently, there is a lack of such articles. Bibliometric analysis enables the assessment of global scientific output regarding sufentanil, aiding decision-making and prioritization of limited resources. This study conducted the first bibliometric analysis of classic articles in the field related to sufentanil, providing clinical practitioners and researchers with insights into the research focal points and evolutionary trajectories within this field.

Sufentanil’s pivotal role in clinical practice has spurred a steady annual update in related research. Out of a total of 1,473 sufentanil-focused articles, China has accounted for 674 publications, indicating a consistent contribution to global anesthesiology literature. As depicted in Figure 3D, we charted China’s annual research output from 2003 to 2023, revealing a systematic initiation of sufentanil research in China following its introduction in 2005, post-FDA approval in 1984. Consistent with our assumption, no articles related to sufentanil were published in China in 2003 and 2004. From 2003 to 2014, although research on sufentanil was updated yearly, there was no significant upward trend. In fact, during this period, the research on sufentanil in most countries maintained an upward or stable trend, with minor fluctuations in publication output in some countries leading to overall variation. After 2015, China introduced the “Supplementary Catalog of Non-Medical Anesthetics and Psychotropic Substances” in accordance with regulations on anesthesia and psychotropic drug control, which included fentanyl substances, including sufentanil. This signifies that sufentanil was officially incorporated into the strict control measures of China’s anti-drug authorities. This regulatory milestone heightened scientific interest, potentially fueling the surge in research observed after 2015. It is also plausible that China’s delayed research onset in sufentanil contributed to this research upsurge. Notably, since 2015, China’s annual sufentanil-related publications have been substantial and escalating, marking a clear upward trend in the field’s research output.

China has distinguished itself as the leading nation in sufentanil research, both in terms of publication volume and academic prestige. This preeminence is largely attributed to the contributions of institutions such as Anhui Medical University, Capital Medical University, and Zhejiang University. The introduction of regulatory measures in 2015 marked a turning point, spurring a surge in the quantity and quality of Chinese publications, which has significantly bolstered the field of sufentanil research. Nonetheless, the modest average citation rate per article underscores the need for future research to balance quantity with quality, striving for innovation and impact. From the collaboration network perspective, China is centrally positioned with thick connection lines to Germany, the United States, and France, representing a very close cooperative relationship. The interconnected global research landscape exemplifies scholars leveraging collective strengths, transcending geographical divides to foster academic synergy.

Employing the Three-Fields Plot analysis in Bibliometrix, we discerned that Liu L from Jiaxing University has a pronounced focus on sufentanil and dexmedetomidine research. A pivotal study demonstrated that dexmedetomidine’s addition to levobupivacaine in transversus abdominis plane (TAP) blocks prolongs analgesia duration and diminishes the need for postoperative sufentanil. Similarly, Xiao F from Jiaxing University has made significant contributions to the study of dexmedetomidine in anesthesia, particularly in cesarean sections. Their work indicates that a 5 μg intrathecal injection of dexmedetomidine can enhance the efficacy of spinal bupivacaine by 24%, coupled with a marked reduction in postoperative sufentanil consumption in the dexmedetomidine group. Both studies highlight the potential of dexmedetomidine to extend analgesia duration when combined with bupivacaine, thereby reducing the reliance on sufentanil, which, despite offering superior postoperative analgesia, is associated with a higher incidence of side effects. The adjunctive use of dexmedetomidine not only mitigates postoperative shivering but also enhances the quality of analgesia in spinal anesthesia for cesarean sections. The strategic co-administration of sufentanil with other anesthetics is posited to attenuate side effects and prolong the analgesic effect, facilitating the judicious selection of anesthetic agents in diverse clinical scenarios (Liu et al., 2019).

Publications on the application of sufentanil in anesthesia have appeared across 355 distinct journals, with the top 10 journals amassing 504 articles, representing 34.21% of the overall total. Notably, within the top 10, 4 are classified in the JCR Q1 category, and 1 in Q2, underscoring the field’s high-impact status. Moreover, 4 of these journals boast an impact factor exceeding 5, reflecting both the field’s rigorous research standards and the significant interest in sufentanil research. The preponderance of anesthesia-focused publications among the top 10 journals aligns with Bradford’s Law, which posits that the majority of specialized literature is concentrated within a select group of core journals, complemented by a scattering in peripheral and generalist journals (e.g., nursing journals) (Desai et al., 2018). This distribution underscores the pivotal role of anesthesia journals as the central platform for disseminating and exchanging research on sufentanil applications in anesthesia.

The 1,473 articles analyzed primarily draw from clinical medicine, with a significant concentration in anesthesia, nursing, pharmacy, cellular biology, and genetics, highlighting a pronounced emphasis on medical and clinical services. As an opioid analgesic frequently utilized in surgical anesthesia, sufentanil has been a subject of extensive research, particularly within the anesthesia discipline. Of the articles reviewed, 656 (44.53%) pertain to anesthesia. The fields of internal medicine and pharmacology also play a crucial role, driving the discovery of improved drug usage protocols and clinical administration strategies to ensure patient safety through effective medication and monitoring. While anesthesia-related articles predominate, the distribution across other pertinent fields is relatively uniform. It is noteworthy that the last four disciplines in the top 10 are specialized areas, including cardiovascular, critical care, obstetrics and gynecology, and respiratory medicine. The top six disciplines are inherently linked to the basic research of sufentanil, indicating a scholarly focus on the drug’s foundational studies from 2003 to 2023. This analysis suggests that sufentanil’s application has been deeply explored within these disciplines. Consequently, clinicians and researchers are encouraged to delve into the optimal utilization of sufentanil in cardiovascular and respiratory medicine, as well as other specialized fields, to enhance therapeutic outcomes.

### 4.2 Research hotspots and frontiers

Over 2 decades, citation bursts among certain keywords have marked periods of intense research activity and new trends. “Fentanyl” surged early, with a citation peak in 2003, underscoring enduring interest in opioid analgesics. “Analgesics opioid” peaked in 2006, reaffirming the significance of opioids in pain management. From 2007 to 2009, “anaesthetic techniques”, “labor analgesia” and “spinal-anesthesia” experienced bursts, reflecting an emphasis on enhancing anesthetic methods and obstetric pain relief. “Lidocaine”, a prevalent local anesthetic, surged in 2010, signaling renewed interest in its applications. “Cesarean delivery” showed a strong citation burst from 2013 to 2017, mirroring trends in obstetric anesthesia. “Meta analysis” peaked in 2016, suggesting a methodological shift toward evidence synthesis to guide clinical practice. “Midazolam” and “management” burst in 2016 and 2017, respectively, with “management” continuing to 2023, possibly indicating an increasing focus on holistic patient care in anesthesia. “Sedation” and “postoperative pain” also surged, the latter into the study’s final year, highlighting the enduring issue of postoperative pain control. “Dexmedetomidine”, a newer sedative, burst in 2019, suggesting growing interest in its anesthetic applications. In summary, the keyword burst map illustrates the evolving research landscape in anesthesiology, shifting from traditional opioids to diverse anesthetic techniques and patient management. The sustained focus on postoperative pain and the rise of agents like dexmedetomidine reflect a commitment to enhancing patient outcomes and safety in anesthesia.

Keyword clustering and timeline analysis have delineated the primary research foci within the anesthetic application of sufentanil, including its pharmacokinetics, application scope, administration techniques, benefits, adverse reactions, and synergistic effects with other medications. The findings affirm sufentanil’s status as an efficacious and relatively safe analgesic and anesthetic, extensively utilized across clinical domains. Sufentanil is particularly valued for its superior analgesic profile and hemodynamic stability, positioning it as a principal agent in total intravenous anesthesia and as an anesthetic adjunct in clinical practice. It also figures prominently in pain management protocols. In keyword clustering, the side effects and adverse reactions of sufentanil, especially its potential to induce pharyngeal spasms and coughing, have garnered considerable attention. Common opioid-related adverse effects encompass nausea (Zhang et al., 2023), vomiting (Qian et al., 2022), respiratory depression (Blackburn, 1987), urinary retention (Kim et al., 2006), and itching (Waxler et al., 2004). Sufentanil, with its lower μ2 receptor affinity compared to morphine and fentanyl, exhibits reduced respiratory depressive effects. Research indicates that sufentanil’s respiratory depression is dose-dependent, progressing from respiratory rate reduction to potential respiratory arrest (Verborgh and Meert, 1999). Notably, the respiratory depressive effects are transient, shorter than its analgesic effects, and readily reversible with naloxone. Sufentanil’s interaction with various opioid receptors in the respiratory tract may predispose patients to coughing. Additionally, as a citrate salt, it can irritate airway C fibers, precipitating coughing (Koppert et al., 2012). The precise mechanism of cough induction by sufentanil during anesthesia, however, remains elusive. Consequently, selecting appropriate intervention strategies is crucial for mitigating or preventing adverse reactions, thereby enhancing the clinical utility of sufentanil in anesthetic practice.

Ensuring or enhancing the safety and efficacy of sufentanil in anesthesia is a key research focus. One of the methods to improve safety and effectiveness is the combination of sufentanil with other appropriate drugs. For instance, co-administration with propofol has been shown to mitigate cough reflexes by relaxing the chest wall and throat muscles (Prochazkova et al., 2017; Sridharan and Sivaramakrishnan, 2019). Administering intravenous lidocaine hydrochloride prior to anesthesia induction also enhances the safety of the procedure by reducing cough reflex incidence (Li et al., 2016). The amalgamation of dexamethasone (Joung et al., 2018), lidocaine (Nath et al., 2018), dexmedetomidine (Tang et al., 2020)with sufentanil has each demonstrated a capacity to suppress cough symptoms. Notably, the combination of sufentanil with long-acting amide local anesthetics, such as bupivacaine and ropivacaine, in subarachnoid anesthesia and labor analgesia has been particularly scrutinized. Studies indicate that sufentanil paired with ropivacaine for labor analgesia can optimize epidural blockade, shorten labor duration, extend maintenance time, lower the cesarean section rate, and decrease adverse reactions, including early postpartum hyperalgesia (Chen et al., 2020). Furthermore, this combination in epidural anesthesia for labor has been associated with reduced cesarean section and postpartum bleeding rates (Zhang et al., 2021).

For basic researchers and clinical practitioners, the safety of drug compatibility in clinical use is an issue that must be seriously considered. Therefore, it is crucial to strengthen evidence-based drug evaluation, quality monitoring, and compatibility stability. As seen in the keyword burst analysis with the growth in intensity of “Meta-analysis”, evidence-based medical evaluation in drug compatibility is vital for ensuring patient safety. Thus, using systematic reviews and meta-analysis methods to conduct in-depth analysis of relevant literature provides a solid evidence base to ensure the clinical safety of the combination of sufentanil with other drugs. For instance, the latest meta-analysis, based on evidence from 32 RCT studies, suggests that the combination of esketamine with sufentanil for postoperative patient-controlled intravenous analgesia can improve postoperative analgesia, alleviate postpartum depression, and reduce the incidence of postoperative adverse reactions (Yao et al., 2024). The compatibility stability of sufentanil with other drugs also requires more in-depth research to assess the interactions between different drugs and the potential compatibility effects to ensure the safety of clinical medication use (Ni et al., 2024). Strengthening quality control measures for drug compatibility is also a matter of great concern, which includes the use of efficient and accurate detection methods such as HPLC-DAD and LC-MS/MS for systematic quality monitoring of drug compatibility stability and compatibility (Yao et al., 2024). This provides a strong technical foundation for the detection of drug abuse in clinical settings and the safety and efficacy of drug use.

Additionally, the therapeutic potential of sufentanil in cancer pain management represents a burgeoning field with scope for further exploration. Preliminary research has already demonstrated the drug’s utility in perioperative analgesia for cancer surgeries and in palliative care for advanced cancer patients (Good et al., 2009). Nevertheless, there is a pressing need for extensive, multicenter clinical trials with larger cohorts to standardize assessment criteria and expand our understanding of its applications in oncological pain management.

### 4.3 Research limitations and prospects

The keyword clustering map, with its color-coded clusters, visually encapsulates the diverse research themes in anesthesiology related to sufentanil. The green cluster highlights the critical nature of effective pain management, the blue emphasizes the array of anesthetic techniques coupled with the necessity for patient monitoring, the red directs attention to the drug’s extensive surgical applications, and the yellow focuses on pharmacokinetics and the paramount concern of patient safety. This analysis captures the extensive scope of sufentanil research, underscoring its role in optimizing anesthesia care and patient outcomes. Looking ahead, future research on sufentanil in anesthesiology should expand upon the established knowledge base and address the limitations of current studies. Drawing from the four major clusters that encapsulate the core research areas, we recommend several avenues for future inquiry.

#### 4.3.1 Applicability of populations and dosage using

Current research has not fully elucidated the optimal administration strategies for sufentanil, especially for special patient populations, such as children and elderly patients, or the differences in the pharmacokinetics and pharmacodynamics of sufentanil in children different patients populations. Studies have indicated substantial variability in the pharmacokinetics and pharmacodynamics of sufentanil among patients, owing to factors such as age, weight, genetic composition, and comorbidities. Pediatric patients, elderly patients, and those with renal or hepatic impairment may require adjusted dosing regimens to avoid adverse reactions. Additionally, the clinical trials of sufentanil in different ethnicities or geographical regions are underrepresented, and the utility and differences in these diverse populations may be a direction that our researchers need to explore in the future. Future research should actively develop personalized dosing strategies based on genetic and phenotypic factors, patient demographics, and comorbid conditions to maximize analgesic effects and minimize side effects. These strategies may involve the use of pharmacogenomics to predict drug responses and optimize dosing on an individual basis. Furthermore, broader clinical trials and long-term studies, including a diverse range of participants, are needed to ensure that dosing recommendations are applicable to different populations and ethnic groups, to understand the impact of chronic sufentanil use, and to establish safe and effective long-term analgesic treatment guidelines. Special attention should be paid to developing age-specific dosing guidelines for pediatric and geriatric populations, as these “vulnerable groups” and may be sensitive to the effects of the drug. Further research should aim to establish age-appropriate dosing guidelines that account for developmental and physiological changes. Utilizing real-world data from large healthcare databases can provide insights into the effectiveness and safety of various sufentanil administration strategies in clinical practice.

#### 4.3.2 Novel drug combinations and non-opioid adjuvants

In light of the increasing concerns over opioid-related adverse events, researchers need to intensify their focus on the use of non-opioid adjuvants in conjunction with sufentanil to improve pain control and reduce opioid dependent. Existing studies have demonstrated the potential of drug synergy to enhance efficacy and reduce the required dosages of opioids, thereby potentially lowering the risks of adverse reactions and dependence. Studies have explored the synergistic effects of sufentanil when combined with adjuvants such as local anesthetics, alpha-2 agonists such as dexmedetomidine, and N-methyl-D-aspartate receptor antagonists suggesting that the combination of sufentanil with non-opioid analgesics, such as acetaminophen and non-steroidal anti-inflammatory drugs, can significantly improve pain control in certain clinical settings. However, these studies were often preliminary and require further validation. Future research should focus on expanding clinical evidence, understanding the mechanisms of action, and assessing long-term outcomes. These endeavors will help establish evidence-based guidelines for the use of these combinations, ultimately enhancing patient care and safety. Additionally, with the significant development of drug delivery systems, investigations of innovative drug delivery systems may offer opportunities for the controlled release of sufentanil and adjuncts, potentially optimizing therapeutic effects and minimizing side effects. Conducting economic evaluations based on Pharmacoeconomics to determine the cost-effectiveness of the combination therapy is also necessary, as the resultant information may influence its adoption in clinical practice.

#### 4.3.3 Technological innovation

Technological innovations have profoundly affected the field of anesthesiology. These advances have enhanced the administration rate, monitoring capability, overall safety and efficacy of sufentanil in clinical practice. For instance, target-controlled infusion of sufentanil offers stable analgesia, improved hemodynamic control over bolus intravenous anesthetic administration, anticipated recovery, and enhanced perioperative anesthetic quality. Advanced monitoring technologies such as bispectral index monitors facilitate the assessment of anesthetic hypnosis components, enabling the optimal titration of sufentanil for maximal analgesic effects. Innovations in drug delivery, such as computer-controlled infusion pumps and patient-controlled analgesia (PCA) devices have also improved the utilization of sufentanil, ensuring the delivery of consistent drug levels and patient comfort.

With the advent of the data era and artificial intelligence (AI), machine learning and AI have been increasingly applied in various medical domains. Their application in anesthesia can be used to construct predictive models for sufentanil demand, enhance patient safety and reduce the need for manual titration. Future research should focus on areas such as nanotechnology, remote monitoring, telemedicine, and pharmacogenomics to enhance the use of sufentanil in the field of anesthesia. For example, nanotechnology-based drug delivery systems can enable the targeted delivery of sufentanil, potentially reducing systemic side effects and improving treatment outcomes. Advances in genomic technology can help predict individual responses to sufentanil, guide personalized anesthesia plans and reduce the risk of adverse reactions. Remote monitoring technology can expand the use of sufentanil to minor procedures or outpatient settings, thereby broadening the scope of anesthesia care. The development of new drug delivery systems, such as transdermal or implantable devices, can enable and controlled release of sufentanil, optimizing pain management and patient comfort. These innovative areas are worthy of attention from future researchers.

## 5 Conclusion

This study offers a visual and tabular synthesis of the literature on sufentanil in anesthesia, encompassing publication trends, research themes, and hotspots. While employing an array of visualization tools for a multidimensional presentation, the study acknowledges its limitations. Notably, the citation analysis is confined to English-language entries within the SCI-Expanded, SSCI, and CPCI-S cores of the WOS database, potentially excluding relevant literature from other databases or languages. Despite these constraints, the research provides valuable insights into future trends, serving as a reference for scholars in this domain, guiding them in strategic direction, team formation, and methodological choices.

## Data Availability

The original contributions presented in the study are included in the article/supplementary material, further inquiries can be directed to the corresponding author.
